# The Cognitive Transformation of Japanese Language Education by Artificial Intelligence Technology in the Wireless Network Environment

**DOI:** 10.1155/2022/7886369

**Published:** 2022-07-07

**Authors:** Su Zhang

**Affiliations:** School of Foreign Languages and International Education, Dalian Ocean University, Dalian 116023, Liaoning, China

## Abstract

This study aims to solve the multiscale problems faced by the current classroom student behavior target detection based on the convolutional neural network (CNN) in the wireless network environment. Firstly, the recent reform of Japanese language education is introduced. Secondly, the multiscale problem research of classroom student behavior target detection is discussed. A CNN-based new extraction network is designed based on dilated convolution and pyramid features. An anchor reconstruction algorithm based on improved K-means clustering is presented for the self-made student behavior dataset. Finally, the performance of the designed algorithm is tested. The anchor reconstruction algorithm's mean average precision is 83.2%, and the average intersection over union is 73.7%. The experimental results of this scheme outperform the original single-shot multibox detector and K-means algorithms. Compared with other algorithms, the designed multiscale detection algorithm of classroom student behavior has the best detection effect on Pascal visual object classes (VOC) dataset. The detection accuracy of the entire dataset is 79.8%. Overall, the multiscale detection algorithm for classroom student behavior has a better detection effect on the Pascal VOC dataset and has good generalization ability and robustness. This research can guide students to recognize their class status and make corresponding adjustments to improve their learning efficiency, which has essential research significance and application value.

## 1. Introduction

In recent years, with the construction of intelligent campuses in critical universities across the country, the smart campus combining teaching, scientific research, management, and the Internet of things (IoT) has been created in the wireless Internet environment, improving the efficiency of campus management and teaching. It is an important topic to change the thinking of Japanese teachers and continuously improve teaching methods through artificial intelligence (AI) empowerment in the AI era [1]. As a powerful auxiliary means of educational informationization, computer vision technology such as object detection is helpful to the improvement of classroom teaching evaluation and teaching management. It is one of the most important research directions in the future.

Students' classroom behavior can fully reflect their learning effect and participation in teaching. Teachers can fully understand the learning effect of students and adjust teaching content and teaching methods through student behavior to improve the teaching quality [2, 3]. Target detection is mainly divided into traditional and CNN-based target detection algorithms [4]. Jiang et al. [5] proposed a multitask target detection model based on CNN. The model processes radar echo data directly, eliminating the need for time-consuming radar signal processing. The detection method can detect and locate targets in the multidimensional space of distance, velocity, azimuth, and elevation by using both time and frequency information. The experimental results showed that, compared with traditional radar signal processing methods and other advanced methods, the CNN-based detector has better detection performance and measurement accuracy in terms of distance, velocity, azimuth, and elevation and has stronger sensitivity to noise. Bai et al. [6] proposed an improved single-shot multibox detector (SSD) model based on deep feature fusion. The SSD algorithm adopted deep feature fusion between the object detection and adjacent feature layers, including convolution kernels and pooling kernels of different sizes, downsampling of low-level features, and deconvolution upsampling of high-level features. The network was improved by combining the target frame recommendation strategy in the SSD algorithm with the frame regression algorithm. The results showed that the improved SSD algorithm improves the detection accuracy and detection rate of targets, and the effect is more obvious for smaller targets.

To this end, this work designs an extraction network based on dilated convolution and pyramid features to solve the multiscale problem. The multiscale problem of classroom student behavior target detection was studied after the current reform of Japanese language education was introduced. The results demonstrate that the cascaded convolution structure with multiple expansion rates can strengthen the multiscale receptive field of the network. In addition, the novelty of the research is that an anchor reconstruction algorithm based on improved K-means clustering is designed to learn and cluster the shapes of all objects in the self-made classroom student behavior dataset. Compared with other existing student behavior detection algorithms, the multiscale detection algorithm of student behavior based on classroom teaching can improve the detection effect of the system. In this paper, Section 1 briefly introduces the background of wireless IoT and AI technology.  Section 2 studies the multiscale problem and target detection for Japanese education reform and students' classroom behavior and constructs the network design structure of feature extraction.  Section 3 improves the anchor point reconstruction algorithm of K-means clustering and conducts data training and experimental verification.  Section 4 analyzes the experimental results, compares the effects of different models, and draws the experimental conclusion. This research has practical application and reference value for developing AI technology in the wireless network environment.

## 2. Materials and Methods

### 2.1. Reform of Japanese Language Education

At present, AI and education are gradually moving towards a deep integration. Teachers in the context of intelligent education need the ability for human-machine cooperation. At present, Japanese teaching in Chinese universities is still dominated by large classes. Teachers usually pay attention to the overall learning status and performance of students and ignore the individual development. Therefore, it is indispensable to introduce AI technology to teaching assistance in Japanese education reform. When designing teaching tasks, teachers should depend on their professional ability and carry out personalized training plans with the assistance of AI [7, 8].

Teachers can quickly understand students' learning status, improve students' learning efficiency, and teach students according to their aptitude through applying AI technologies such as face recognition technology, speech recognition technology, and machine translation to teaching. Speech recognition technology can assist in guiding students' Japanese pronunciation and lighten the teaching burden of teachers so that they have more time to guide students' Japanese oral expression. However, large classes of 45 minutes (about 30 students) do not allow each student to read the text completely, and teachers do not have enough time to carefully correct each student's pronunciation. Voice recognition technology will change this awkward situation. Through the hardware and software equipment of the smart classroom, and the built-in MP3 as the benchmark, the whole class reads the text simultaneously, and the speech recognition technology can mark the mistakes in the students' pronunciation at any time. The teacher can clearly understand and control the pronunciation of each student at the terminal and provide learning guidance for each student in time through the information and data feedback of the AI system. In the process of Japanese interpretation, the AI system can perform scene reproduction, animation display, listening and speaking sample data storage, interpersonal interaction data recording, and human-computer interaction during user training. The penetration of educational technology or the display of stereoscopic images can stimulate students' enthusiasm for learning and further improve their learning efficiency [9].

### 2.2. Research on the Multiscale Problem of Classroom Student Behavior Target Detection

Object detection models based on CNNs are classified into the one-stage type based on region proposal and the two-stage type based on regression [10, 11]. The one-stage type based on region proposal directly uses the forward neural network to predict the prediction box of interest. The regression-based two-stage type first generates proposal boxes and then refines the regional features extracted from deep neural networks. However, both methods face the multiscale problem. Changes in the size of the object detection instance will affect the final effect of the detection. Multiscale image pyramids are an intuitive way to solve multiscale problems. However, the image pyramid method increases inference time and is therefore not suitable for real-time applications. The image of each layer of the Gaussian pyramid is used to subtract the predicted image after upsampling and Gaussian convolution of the image of the previous layer, generating a series of different images, that is, decomposed images. In the classroom student behavior target detection scene, cameras are set on both sides of the classroom blackboard to record the classroom situation of the students and then select clear still picture frames as the data set [12]. Because the distance between each row of students and the camera is different, the size of the students in the front row is large, and the size of the students in the back row is negligible. When the classroom is large, and the number of students is large, the size of the students in the front row is 3–5 times that of the students in the last row. In addition, the current object detection algorithms are all detected on Pascal visual object classes (VOC) dataset or the Common Objects in Context (COCO) dataset. Both datasets involve objects in natural scenes. If a series of regional candidate frames are established according to the size of the natural scene target to detect the student behavior target, it will inevitably lead to the candidate frame not matching the actual target, resulting in a further reduction of the robustness of the model. Ultimately, this results in suboptimal object detection for classroom students.

### 2.3. Design of Network Structure

#### 2.3.1. Network structure

**Figure 1:**
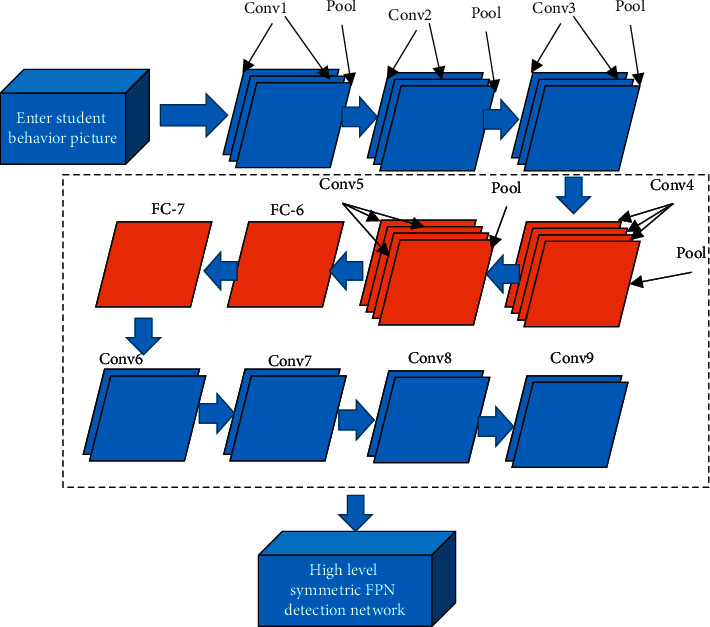
Basic feature extraction network.

The multiscale target detection network for classroom learning behavior includes a basic feature extraction network and high-level symmetric feature pyramid networks (FPN). The structure of the basic feature extraction network is shown in Figure 1. The first five groups of convolutional layers in the visual geometry group (VGG)-16 network are sequentially inputted into classroom learning images. Then, fully connected layers 6 and 7 are replaced with corresponding convolutional layers. The fully connected layer eight and the softmax classification layer are discarded. Finally, the remaining network adds four additional groups of convolutional layers at the end as the basic feature extraction network to realize the transformation of student behavior goals from the image domain to the feature domain.

**Figure 2:**
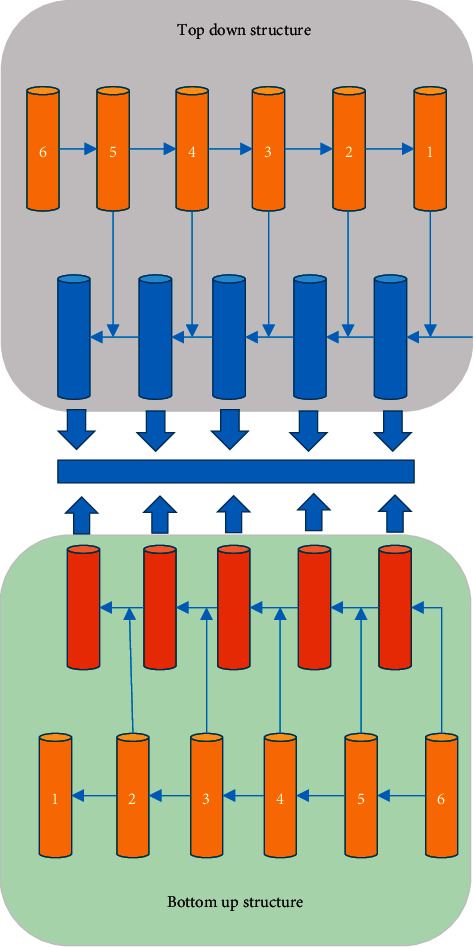
Structure of high-level symmetric FPN.

In Figure 1, the students in the front row of the classroom and the students in the back row are of different sizes. This study designs a multiscale symmetric FPN including both top-down and bottom-up, as shown in Figure 2. The network generates the position information of the prediction box and the corresponding classification probability of the student behavior target. The original FPN structure only transfers the information of the highest-level feature map to the lower-level feature map, and the highest-level feature map does not add information about the low-level feature map [13, 14]. After a high-level symmetric FPN detection network, each predicted feature map contains the other five-dimensional feature maps' detailed information and semantic information.

#### 2.3.2. Design of Multiscale Dilated Convolutional Layers

**Figure 3:**
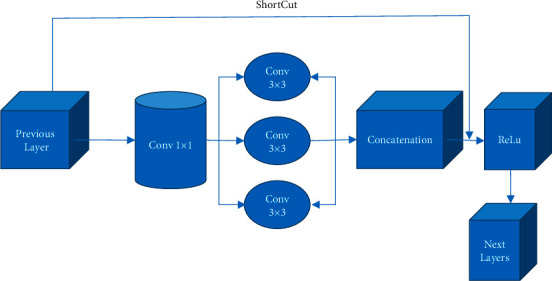
Cascaded multidilation rate convolution structure with residual units.

Dilated convolution can increase the size of the convolution kernel and increase the receptive field without changing the weight of the original convolution kernel. Dilated convolutions have been extensively used in semantic segmentation to fuse large-scale contextual information through dilated convolutions [15, 16]. The feature maps generated by dilated convolutions with different dilation rates are parallelized so that the neurons in the output feature map contain receptive fields of multiple scales, thereby improving the detection effect of classroom students' behavioral targets. In addition, the propagation of gradients in multisized receptive fields tends to diverge, making it difficult for the network to converge. Therefore, a residual unit is added to solve this problem.  Figure 3 reveals the specific network structure.

The principle of the residual unit is shown in the following equation:

(1) $$\begin{array}{c} x_{L}=x_{l}+\sum_{i=l}^{L-l}F\left(x_{i},w_{i}\right), \end{array}$$

where *x*_*L*_ represents the *L*th layer output of the residual unit; *x*_*l*_ represents the *lth* layer input of the residual unit; *x*_*i*_ represents the *ith* feature layer; *w*_*i*_ represents the corresponding convolution weight of the *ith* feature layer; *F* (*x*_*i*_, *w*_*i*_) represents the convolution operation on *x*_*i*_ and *w*_*i*_. The information of different deep networks can be fully integrated through the shortcut connection to meet the accuracy requirements of classroom student behavior target detection.

### 2.4. Region Candidate Box Reconstruction Algorithm

#### 2.4.1. Anchor Mechanism

The regional candidate frame generation mechanism can also be called the anchor mechanism, generating regional candidate frames. The generation of region candidate boxes directly affects the direction of target feature learning [17]. The introduction of the anchor mechanism reduces the repeated calculation of features and improves the detection speed and accuracy to a certain extent. However, the real-time performance of the algorithm is relatively poor due to a large number of calculations. SSD is a real high-quality, high-precision real-time detection algorithm. The SSD algorithm predicts candidate boxes on feature maps with different resolutions, directly divides *S* ×*S* grids according to their relative proportions to the original image, and generates candidate boxes of increasing sizes in the center of each grid [18, 19]. After the SSD algorithm obtains the region candidate frame, the probability of objects contained in these boxes is predicted, and the position offset of the box is calculated simultaneously. Then, the nonmaximum suppression algorithm filters out the candidate frame most likely to contain the target and readjusts the candidate frame in combination with the position offset of the frame to obtain the final detection result [20, 21].

#### 2.4.2. Analysis of the Scale of Classroom Student Behavior Datasets

Selecting the appropriate size of candidate boxes for the classroom behavior dataset and maximizing the dispersion of candidate boxes of different scales and widths to comprehensively cover the dataset can improve object detection results' effectiveness. Each candidate box size can be regarded as a cluster center of a small unit. Each small unit is a network optimization unit. Therefore, the classification and regression prediction parameters are optimized according to the corresponding training set. The core idea of the traditional *K*-means clustering algorithm is for a given data sample. The distance is used as the classification standard to divide the data within a short distance into a cluster. The number of clusters K can be set manually according to different needs [22, 23]. The specific process of the *K*-means clustering algorithm is as follows.

First, *k* cluster centers are randomly selected from the sample data set *X*, defined as set *C*, as shown in the following equation:

(2) $$\begin{array}{c} C=\left\{c_{1},c_{2},\ldots ,c_{k}\right\}, \end{array}$$

where 1 < *k* ≤ *n*, and *n* represents *n* objects.

Second, the Euclidean distance of all sample data to each cluster center is calculated. Then, the samples are divided into clusters corresponding to the nearest cluster centers [24]. Equation (3) describes the Euclidean distance.

(3) $$\begin{array}{c} d\left(x_{i},c_{j}\right)=\sqrt{\sum_{q=1}^{m}{\left(x_{iq}-c_{jq}\right)}^{2}}, \end{array}$$

where *x*_*i*_ represents the *ith* sample; *c*_*j*_ represents the *jth* cluster center; *d*(*x*_*i*_, *c*_*j*_) represents the Euclidean distance from the *ith* sample to the *jth* cluster center.

Third, the center of each cluster is calculated according to the centroid method. Equation (4) defines the *jth* cluster center *c*_*j*_.

(4) $$\begin{array}{c} c_{j}=\frac{\sum_{x_{i}\in S_{t}}x_{i}}{\left|S_{t}\right|}, \end{array}$$

where *S*_*t*_ represents the number of objects in the *jth* cluster.

Fourth, the last *k* cluster centers are compared to determine whether the center has changed. If the center changes, skip to the second step for the next iteration; otherwise, the algorithm converges and outputs *k* clusters.

#### 2.4.3. Anchor Point Reconstruction Algorithm Based on Improved K-Means Clustering

The anchor reconstruction algorithm uses the width *w* and height *h* of all ground-truth boxes as input samples in the classroom behavior dataset. According to the distance metric, all the width and height samples are divided into categories corresponding to their nearest shape clustering centers. In this way, the distance between target candidate frames with significant shape differences is as considerable as possible, and the distance between target candidate frames with a similar shape is as tiny as possible. Here, a new similarity distance metric, the intersection distance metric over the union, is employed, as shown in the following equation:

(5) $$\begin{array}{c} d\left(\text{Other,Center}\right)=1-IoU\left(\text{Other,Center}\right), \end{array}$$

where *d*(Other,Center) denotes the distance between other borders and the cluster center border; *IoU*(Other,Center) represents the intersection ratio of other borders and the cluster center border, as shown in the following equation:

(6) $$\begin{array}{c} IoU\left(\text{Other,Center}\right)=\frac{S\left(\text{Other}\cap \text{Center}\right)}{S\left(\text{Other}\cup \text{Center}\right)}, \end{array}$$

where *S*(Other∩Center) denotes the area of the intersection of the two borders; *S*(Other ∪ Center) signifies the area of the union of the two borders.

All the width and height samples are randomly selected from the *k* cluster center points and performed initial division. These central stores are then iteratively optimized until width and height clustering results are obtained for all student behavioral targets. The genetic algorithm is used for the initial center point of the global optimization algorithm [25]. The fitness function in the genetic algorithm is a crucial part of the anchor reconstruction algorithm. Each anchor box in the study is a training sample. Each anchor box is either marked as a background or associated with an actual edge box, generating many anchor boxes and producing many negative class samples. Equation (7) describes the fitness function designed here.

(7) $$\begin{array}{c} f=\frac{{\bar{D}}_{\text{between}}\left(d\left({\text{Center}}_{i},{\text{Center}}_{j}\right)\right)}{1+{\bar{D}}_{in}\left(d\left(x_{i}^{\left(j\right)},{\text{Center}}_{j}\right)\right)},\\ {\bar{D}}_{\text{between}}\left(d\left({\text{Center}}_{i},{\text{Center}}_{j}\right)\right)=\frac{2}{k\left(k-1\right)}\sum_{i=1}^{k-1}\sum_{j=i+1}^{k}d\left({\text{Center}}_{i},{\text{Center}}_{j}\right),\\ {\bar{D}}_{\text{in}}\left(d\left(x_{i}^{\left(j\right)},{\text{Center}}_{j}\right)\right)=\frac{1}{k}\sum_{j=1}^{k}\sum_{i=1}^{n_{j}}\frac{d\left(x_{i}^{\left(j\right)},{\text{Center}}_{j}\right)}{n_{j}}, \end{array}$$

where *d*(*Center*_*i*_, *Center*_*j*_) represents the intersection ratio distance between the *ith* and the *jth* cluster center; *d*(*x*_*i*_^(*j*)^, *Center*_*j*_) represents the intersection ratio distance between the *ith* sample of the *jth* class and the *jth* cluster center; *n*_*j*_ represents the *jth* class total number of samples.

**Figure 4:**
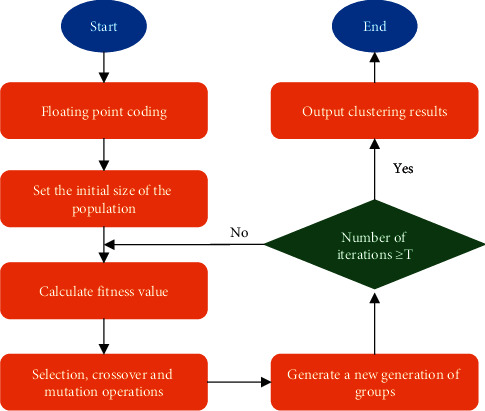
Flow of the anchor reconstruction algorithm based on improved K-means clustering.

Moreover, the classification and offset of each anchor box are labeled. Suppose an anchor box A is assigned a ground-truth bounding box B. On the one hand, the class of anchor box A will be marked the same as B. On the other hand, the offset of anchor box A will be marked according to the relative position of the center coordinates of B and A and the relative size of these two boxes. Given the different positions and sizes of different boxes within a dataset, the model can apply a transformation to those relative positions and dimensions to get an evenly distributed, easily adaptable offset.  Figure 4 displays the flow of the anchor reconstruction algorithm designed here.

### 2.5. Experimental Configuration

This experiment trains and validates the multiscale detection method of classroom student behavior based on a deep learning framework and makes a data set of student behavior in class for model verification and evaluation. The study tests five thousand test images. In addition, the generalization ability of the model has been verified on the public Pascal VOC dataset. The 2,501 images in Pascal VOC2007 and 5,717 in Pascal VOC2012 are used as the training set, and the 4,952 images in Pascal Voc2007 are used as the test set. The specific environment configuration is a central processing unit Intel Core i7-6700 and 4 Graphic Processing Units. The model number is GTX 1080Ti. The operating system is Windows 10, and the programming language is *Python* 2.7, Matlab 2014b.

In addition, the slope of the LeakyReLU activation function in the detection network is set to 0.2; the momentum is 0.9; the batch size is 16; the times of training are 90; the initial learning rate is 0.001. After 50 iterations, the learning rate declines to 10^−4^ by a ratio of 0.1. At 70 iterations, it is reduced to 10^−5^ by a ratio of 0.1. Throughout the training process, the model learning rate decays to 0.0005. Average precision (AP) is used to measure object detection accuracy. Frames per second are a measure of the speed of object detection. The average intersection of the models is used to measure how well the predicted box matches the ground truth. The parameter Mb is used to measure the space complexity of the model.

## 3. Results and Discussion

### 3.1. Comparison of Data Enhancement Effects

**Figure 5:**
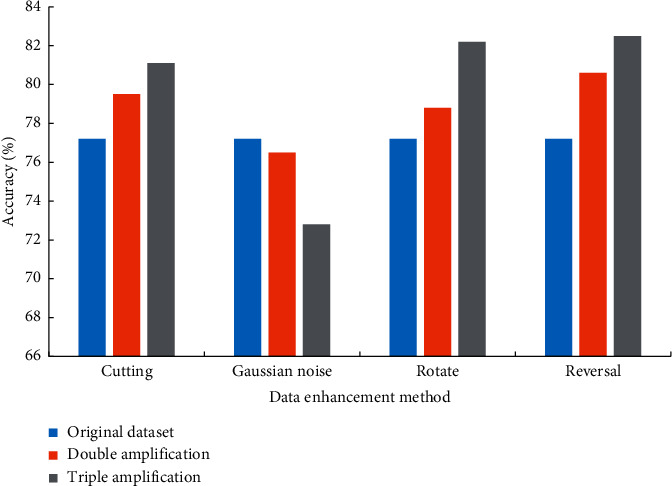
Comparison of the effects of four data augmentation techniques.

The diversity and complexity of training samples play an essential role in the improvement of the model detection effect and the improvement of the generalization ability of classroom students' behavioral goals. Here, data augmentation methods are used to improve the overall performance of model training. Four data augmentation methods, flipping, cropping, rotating, and adding Gaussian noise, are analyzed to generate new sample numbers. The number of samples is one and three times the initial training set for comparative experiments. The comparison results of the four data enhancement techniques are shown in Figure 5.

**Figure 6:**
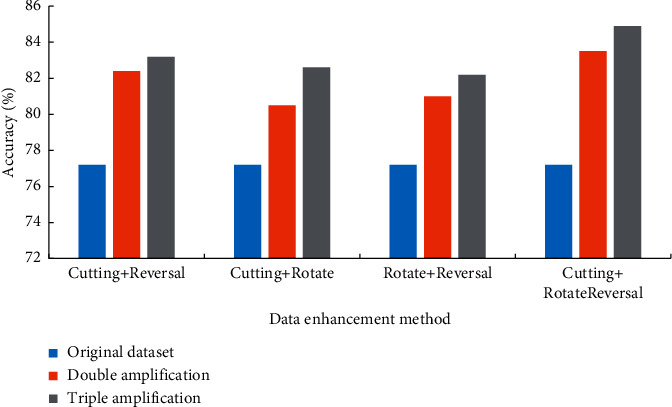
Comparison of combined effects of flip, crop, and rotate methods.

As can be seen from Figure 5, data expansion and enhancement techniques are mainly divided into cropping, rotation, and Gaussian noise filtering. Among them, the three methods of flipping, cropping, and rotating can improve the accuracy of the trained model. Before the cropping method is adopted, the recognition accuracy of the data enhancement technology is only 77.5%. After the double enhancement of the cropping method, the recognition accuracy of the data expansion technology can be improved to 79.8%; the recognition accuracy of the data expansion can be improved to 81.5% under the cropping method after the triple enhancement. In addition, the model recognition accuracy is 77.8% before using flip technology to enhance the data expansion effect; after double-enhancing using flipping technology, the model recognition accuracy reaches 81.8%; the model recognition accuracy after triple-enhancing attains 83%. The higher the training samples, the better the effect. In addition, Gaussian noise will reduce the recognition effect of the trained model to a certain extent. Therefore, the combined effects of flipping, cropping, and rotating methods are further tested for comparison, as presented in Figure 6.

In Figure 6, the hybrid method of flipping, cropping, and rotating has the best effect. When the amount of data is doubled, the accuracy rate is 83.5%; when the amount of data is tripled, the accuracy rate is 84.9%. Therefore, in all experiments, the training data are in the form of a combination of three methods of flipping, cropping, and rotating, and the data set is tripled to obtain the best detection effect for classroom student behavior.

### 3.2. Comparison of Anchor Clustering Results

**Figure 7:**
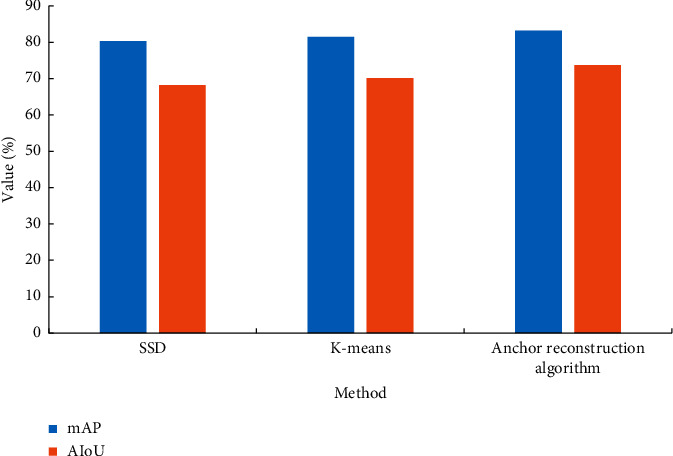
Comparison of three anchor generation algorithms.

The original SSD algorithm, the traditional K-means algorithm, and the anchor reconstruction algorithm are compared.  Figure 7 presents the comparison results of the three algorithms.

In Figure 7, the MAP of the designed anchor reconstruction algorithm is 83.2%, and the AIoU is 73.7%, which is better than the original SSD algorithm and K-means algorithm in both MAP and AIoU. The anchor reconstruction algorithm can overcome the sensitivity of the K-means algorithm to the initial cluster centers. The shape of the predicted candidate box represented by the clustering results can better fit the real shape of the target on the classroom student behavior dataset. The robustness has been dramatically improved compared with the traditional K-means and the original SSD algorithms.

Performance analysis of multiscale detection algorithm of student behavior in the classroom.

**Figure 8:**
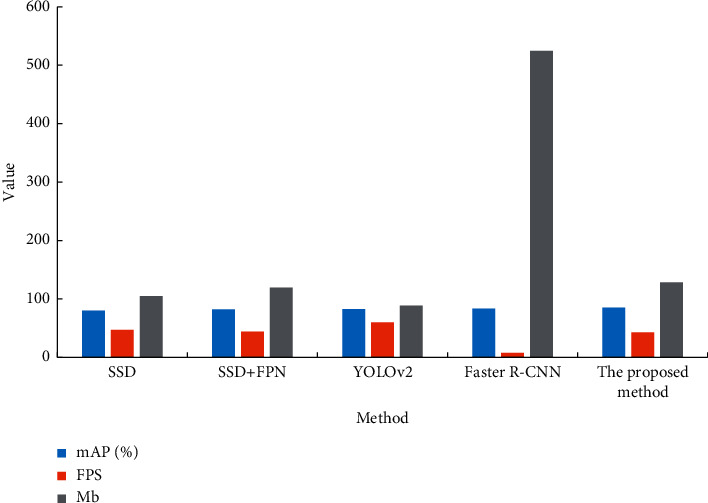
Performance comparison of different algorithms on the classroom student behavior dataset.

The SSD, SSD + FPN, YOLOv2, and faster R–CNN algorithms are selected for inspection and comparison with the proposed multiscale detection algorithm for classroom student behavior. The specific detection results are shown in Figure 8.

Figure 8 suggests that the multiscale detection algorithm designed for classroom student behavior is significantly better than other algorithms in terms of all-around performance. The detection speed of the multiscale detection algorithm of student classroom behavior is not much different from that of the SSD algorithm and SSD + FPN. However, this algorithm has an accuracy of 85.1%, higher than other algorithms. This is because it adopts parallel dilated convolution and symmetric FPN, enabling the network to fully integrate the information of different depth networks, which is conducive to detecting student behavioral targets. In addition, the model using the faster R–CNN algorithm has the highest system data transfer rate, far exceeding the other several detection models.

**Figure 9:**
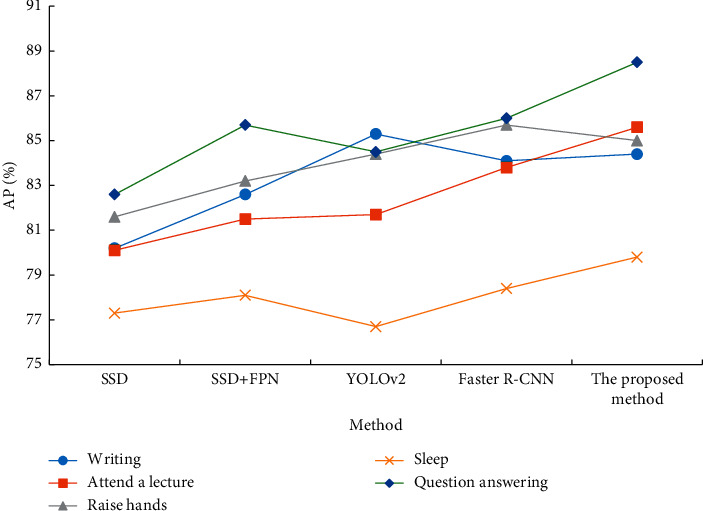
Comparison of the detection effects of different algorithms on five types of actions: writing, listening, raising hands, sleeping, and answering questions.

The comparison of the detection results of the five detection algorithms for the five types of actions of writing, listening, raising hands, sleeping, and answering questions is shown in Figure 9.

In Figure 9, the AP values of the multiscale detection algorithm reported here are 84.4% for writing, 85.6% for listening, 87% for raising a hand, 79.8% for sleeping, and 79.8% for answering. The detection AP value of the problem is 88.5%. The detection effects of listening, raising a hand, sleeping, and answering questions are better than other algorithms. From the overall trend, since raising a hand has prominent behavioral characteristics, the detection effect of this behavior is relatively good. On the contrary, the sleeping behavior is easily blocked by other behaviors, so the detection effect is poor.

**Figure 10:**
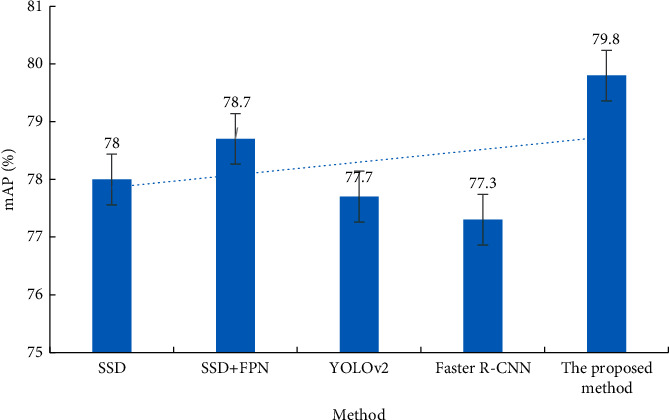
Comparison of detection results of different algorithms on Pascal VOC dataset.

The algorithm proposed here is trained and tested on the public Pascal VOC dataset to verify its generalization ability and effectiveness.  Figure 10 presents the detection MAP results of the five algorithms on the Pascal VOC dataset.

In Figure 10, compared with other algorithms, the multiscale detection algorithm of classroom student behavior has the best detection effect on the Pascal VOC dataset. The detection accuracy of the entire dataset is 79.8%. Overall, the multiscale detection algorithm for classroom student behavior shows excellent generalization ability and robustness.

To sum up, the recognition effects of students' classroom behavior of the algorithm proposed here are better than other algorithms. The average recognition accuracy of several algorithms is analyzed. The AP value of the algorithm proposed here is 84.4% for writing, 85.6% for listening, 87% for raising hands, 79.8% for sleeping, and 79.8% for answering. On the Pascal VOC dataset, the detection accuracy of the algorithm is 79.8%. Overall, the multiscale detection algorithm for classroom student behavior is superior to the other algorithms regarding detection effect and robustness.

## 4. Conclusions

The detection of student behavior in the classroom environment combines classification tasks and positioning tasks, reflecting students' participation in classroom teaching and playing a vital role in promoting Japanese language education and teaching informatization. Aiming at the multiscale problem in which the size of the students in the front row and the back row is quite different in the classroom scene, the network structure and the reconstruction of the candidate frame size are mainly studied. A multiscale detection network is designed based on the SSD algorithm for the network structure under the multiscale problem. The FPN with a symmetric system is designed for the shortcoming of the lack of shallow detail information in the deep feature map of FPN. In addition, a candidate box reconstruction algorithm based on improved K-means clustering is designed to obtain the width and height dimensions of the true distribution of the dataset. The research results demonstrate that the anchor reconstruction algorithm has greater robustness than the traditional K-means algorithm and the original SSD algorithm. The algorithm is superior to other algorithms in the comprehensive performance of the multiscale detection algorithm of student behavior in the classroom and has good generalization ability and robustness. The disadvantage is that the designed classroom student behavior detection algorithm does not have an advantage in the number of model parameters. Therefore, the detection algorithm can be further optimized from the perspective of model compression and network lightweight in the future.

## Data Availability

The data underlying the results presented in the study are available within the manuscript.
